# Distribution Patterns of Degeneration of the Lumbar Spine in a Cohort of 200 Patients with an Indication for Lumbar MRI

**DOI:** 10.3390/ijerph19063721

**Published:** 2022-03-21

**Authors:** Philipp Näther, Jan Felix Kersten, Ingmar Kaden, Kemal Irga, Albert Nienhaus

**Affiliations:** 1 Unfallkrankenhaus Bergmannstrost Halle (Halle Trauma Centre), Diagnostic Imaging and Interventional Radiology Clinic, 06112 Halle, Germany; ingmar.kaden@bergmannstrost.de (I.K.); kemal.irga@bergmannstrost.de (K.I.); 2 Competence Center for Epidemiology and Health Services Research for Healthcare Professionals (CVcare), Institute for Health Services Research in Dermatology and Nursing (IVDP), University Medical Center Hamburg-Eppendorf (UKE), 20246 Hamburg, Germany; j.kersten@uke.de (J.F.K.); albert.nienhaus@bgw-online.de (A.N.); 3 Department for Occupational Medicine, Hazardous Substances and Health Sciences (AGG), Institution for Statutory Accident Insurance in the Health and Welfare Services (BGW), 22089 Hamburg, Germany

**Keywords:** low back pain, magnetic resonance imaging (MRI), lumbar disc degeneration, age-related changes in lumbar spine, spondylosis, osteochondrosis

## Abstract

Lower back pain is one of the most common causes of a reduced quality of life. Magnetic resonance imaging (MRI) is the best suited imaging technique to detect causes of that pain. We retrospectively evaluated the MRIs of the lumbar spine for 200 patients in order to describe the distribution of signs of degeneration with regard to age, sex, and position of the disc affected. The number of spinal segments affected by degeneration increased with age, as did the number of signs of degeneration per segment. In patients aged between 21 and 30, 38.8% of discs were affected, while for patients aged between 51 and 60, 91.6% of discs were affected. There was no statistically significant gender difference. The lower two segments were most commonly affected by degeneration. The most common were structural changes to the discs, which affected 88.4% of patients over 50. Spondylosis was the most common bone-related change, found in 60.4% of patients over the age of 50. A reduction in disc height increases the likelihood of structural changes to the disc and bone-related changes. When investigating risk factors for developing disc-related diseases, the complex disc degeneration patterns described here should be taken into account.

## 1. Introduction

Lower back pain is one of the most common musculoskeletal disorders requiring diagnosis and treatment [1,2]. It commonly causes a considerable decrease in quality of life, while lumbago also has a significant economic impact. A recent systemic review of direct and indirect costs of back pain in industrialized countries showed costs of 259$ million in Sweden up to a maximum of 71.6$ billion in Germany. Direct cost estimates for the US range from 39$ billion to 126.2$ billion [3].

In Germany, employees have the right to claim a disability pension beyond the scope of statutory accident insurance if an occupational disease is responsible for the relevant disability. In order for such an occupational disease to be recognized in the case of work-induced lumbar spine degeneration, the diagnosis must state lumbar segment degeneration resulting from disc damage due to high, repeated stress on the back over a long period of time due to heavy lifting and carrying, or due to working in a bent position. In order to standardize the very difficult process for providing this required evidence, a guideline for the assessment of disc-related occupational diseases of the lumbar spine was developed at a consensus conference in 2005. This guideline comprises both exposure criteria (workplace history) and the results of the physical examination and radiological diagnostics.

During the consensus conference, there were uncertainties in the evaluation of the imaging aspects of the assessment. As a result of a lack of, out-of-date, or highly contradictory study results, no consensus was found for some aspects. The classification of individual patterns of findings determined via morphological imaging remained unclear [4].

In order to resolve this, the employer’s liability insurance associations initiated a cross-sectional study of women who have to lift heavy loads as part of their work as compared with women of the same age who do not have to lift heavy loads. Patient transfer in healthcare was determined as a typical load. There are numerous studies on the risk of lumbago (lower back pain) in nursing staff but only very few studies that investigated disc degeneration in nursing staff [5]. In addition to that, the links between disc degeneration, concomitant bone-related disorders, and facet joint arthritis have not yet been sufficiently investigated.

In order to develop an evaluation algorithm for the cross-sectional study, we carried out a retrospective analysis using MRIs of the lower back from the database of a participating site. The results are provided below. Furthermore, we investigate how consistently a radiological classification can be conducted by partly using subjective assessment criteria.

### 1.1. Disc Degeneration

Lumbar degenerative disc disease is a multifactorial process with a highly individual onset and development. The latest studies show that genetic factors have a major impact on the occurrence and severity of lumbar disc and segment degeneration. In addition, other factors such as changes in diet and lifestyle, as well as occupational strain, must be taken into account [6,7,8,9,10].

The most common cause of lower back pain is the degeneration of the lumbar segments. A spinal segment describes the structural and functional unit of vertebrae, discs, and facet joints. In terms of the cause of pain, the focus is on the disc and its degeneration [11].

Degenerative processes begin in the most vulnerable biomechanical element of a segment—the disc [12]. The structural tissue lesions of the cartilage are highly complex. Structural changes to the disc cartilage are the first to appear. This includes the breakdown of the proteoglycans and increasing loss of fluid. As the disorder progresses, the disc loses height [13,14]. Herniation of parts of the disc (nucleus pulposus) may also occur. This can cause pain or neurological problems due to the compression of spinal nerves.

With the progressive degeneration of the disc, compensatory reactions may occur in the surrounding bone. Those include ventral or dorsal bone spurs which increase the surface of the facies intervertebralis. This can be seen as a way of adaptation to the degeneration of the disc. Moreover, there are changes in the facies intervertebralis itself with oedema and, later, increased sclerosis [15]. It is possible that a chronic, abacterial inflammation process is the cause of this.

Osseous changes in general are more frequently observed with increasing age. These lesions correlate topically with the observed disc degeneration processes [16,17]. Another aspect of lumbar segment degeneration is the facet joints that show analogous patterns of arthritic lesions. These are partly associated with extensive hypertrophy of the joint capsules. There is a close link between disc degeneration, changes in the surrounding vertebrae, and facet joint osteoathritis [18,19].

In addition to spinal stenosis as a result of a large-scale disc extrusion, this may also be caused by a combination of disc bulging and hypertrophy of the ligamenta flava. Typically, this spinal stenosis can be found in older patients because it is caused by other advanced degenerative processes [20,21].

On the basis of the existing research, the disc is viewed as the most important element in segment degeneration. This process often begins early on in life. Initial morphological changes can often already be detected in the third decade of life. In order to detect initial degeneration processes, it is therefore essential to include younger subjects in the study, too [12].

Magnetic resonance imaging (MRI) has become widespread in recent decades for the morphological imaging of the lumbar spine because it not only facilitates the evaluation of the ligaments and connective tissues but also of the vertebrae. MRIs also make it possible to depict the myelon and spinal nerves. Because it does not generate any radiation, there is also no limit on the frequency with which MRIs can be conducted.

As a result of the changes to individual anatomical structures in degenerative processes, there are typical patterns of lesions in the MRI (disc signal loss, decreased height, herniation of disc material). In order to make the extent of the lesions objectifiable and comparable, various classification models have been developed [22].

According to pathophysiological models, progression is likely with increasing age. The determining factor for prevalence and extent of degenerative changes is therefore age [9,15]. Having said that, it is important to note that multiple genetic and lifestyle factors also influence how quickly the degenerative processes progress. Those influencing factors include a sedentary lifestyle, an unhealthy diet and secondary consequences like obesity and the related metabolic syndrome [8]. As a result, it is very likely that progression of degenerative processes will vary widely between individuals.

According to the latest biomechanical findings, the degenerative process starts in those segments positioned between the more mobile and more static areas of the spine. These ‘connective segments’ are under particular strain. The lumbosacral transitional discs relating to such a connective segment are L5/S1. Earlier studies showed that the segments most commonly affected by degenerative disc disease are L4/5 and L5/S1 [23,24]. As the disease progresses, changes may start to occur further up in the cranial segments of the lumbar spine.

Previous trial results showed occasionally contradictory findings with regard to the influence of age on the frequency of disc herniation. There are some indications that herniation is more common with increasing age. Other studies show that disc herniation is most common in the 30–59 age group and then declines again [25,26,27].

### 1.2. Study Goal

The aim of this study was to analyze potential evaluation protocols for the assessment of the lumbar spine with regard to segment degeneration. At the same time, it will analyze typical patterns of lesions associated with lumbar segment degeneration as detected by morphological imaging via MRI. Potential correlations with disc, bone, and ligament changes should also be identified. The analysis focused on age groups in order to discover any evidence of trends or the potential chronological sequence of degenerative processes. Another aspect of the study analyzed gender-specific differences in lumbar segment degeneration over time, and by location.

To assess the imaging data we used the following individual criteria:

- Standardized and absolute disc height;
- Presence of spondylosis or dorsal spondylosis;
- Changes in the facies intervertebralis;
- Evidence of disc herniation;
- Signal characteristics and disc structure;
- Nerve compression;
- Spinal stenosis.

## 2. Materials and Methods

Image data were made available by the Institute of Radiology and Neuroradiology at the BG-Klinikum Bergmannstrost (Halle/Saale, Germany) Occupational Accident Clinic for the retrospective study. The clinic is a German national trauma center. The clinics for neurosurgery, trauma surgery, and reconstructive surgery, as well as the center for orthopedics and spinal injury, treat a wide variety of spinal symptoms. Around 550 MRIs are conducted of the lumbar spine every year.

Prior to the start of the study, the study plan was submitted for appraisal to the Halle/Saale Ethics Committee at the Saxony-Anhalt Medical Association. The Medical Association voted in favor on 9 September 2018.

A total of 200 patients were selected for analysis with an indication for lumbar MRI. According to German legal medical practice all patients had given their written consent to the imaging. The range of indications included unspecific lumbar pain, trauma, and radicular symptoms. We attempted to recruit equal numbers of men and women and to achieve an even age distribution across four decades (Table 1). This resulted in eight cells on the table of cohorts. Each cell was to represent 25 patients, e.g., 25 women aged between 21 and 30. These parameters resulted in the sample size of 200 patients.

Inclusion criteria were a patient age from 21 to 60, as well as a complete examination in accordance with a standardized MRI protocol.

Exclusion criteria included having undergone prior surgery on the lumbar spine. Furthermore, patients with active infection/inflammation of the lumbar spine or sequelae resulting from such an infection or inflammation were also excluded. We also excluded patients with isthmic spondylolisthesis. Patients with traumatic lesions in the lumbar spine were also excluded as these are likely to fasten the “natural” course of segment degeneration. As we lack any clinical data on the patients due to the design of the study, we could not include any of those in the criteria for exclusion or inclusion.

The study population comprised a total of four age cohorts (Table 1). An identical number of patients with equivalent sex distribution was included in each age category.

In order to select the patients, we reviewed the imaging data from the clinic from April 2017 backwards. Patients were enrolled in the individual age cohorts in accordance with the inclusion criteria until the intended number of patients for that cohort had been found.

**Figure 1:**
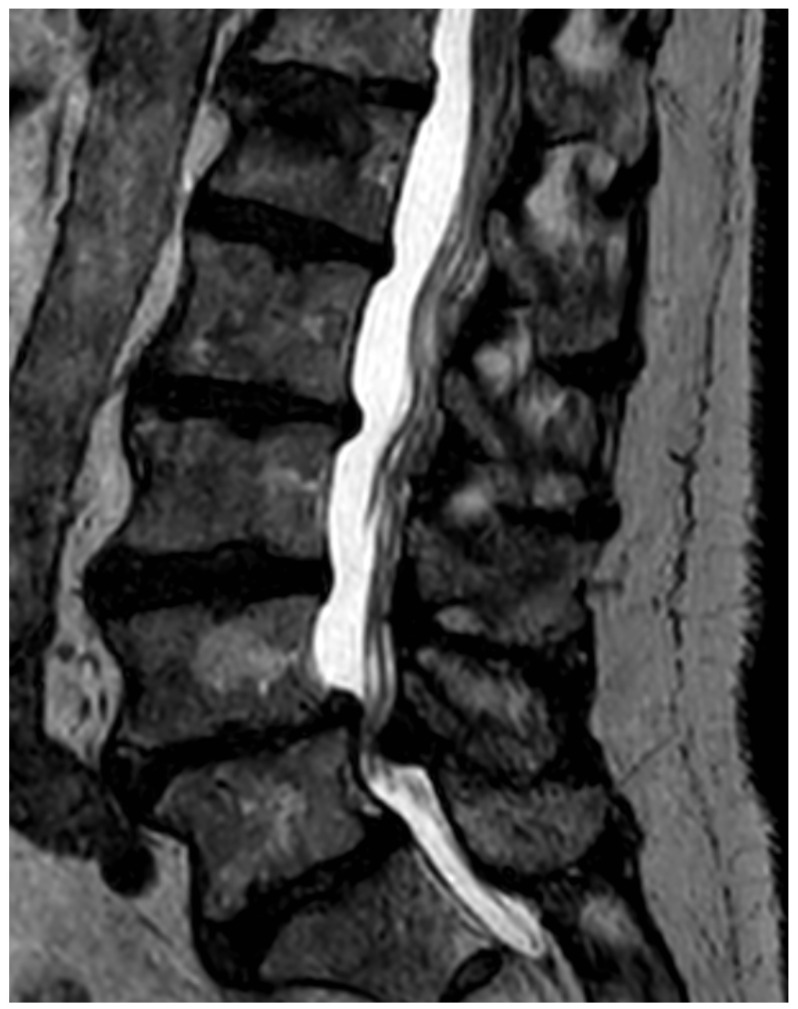
Typical sagittal T2 sequence of a lumbar MRI with signal-attenuated and reduced-height intervertebral discs, evidence of multisegmental disc bulging and degenerative spinal stenosis in segment L4/L5. Additionally, typical vertebral haemangioma at L4.

After the patient list was completed, the MRI images were assessed by a radiologist with several years of experience in reading MRIs of the spine. Figure 1 shows a typical sagittal, T2-weighed image of the lumbar spine with advanced signs of degeneration. 

To classify the in the introduction mentioned evaluation criteria we used the following methods. In order to determine the standardized disc height, the Hurxthal method was used and the disc height given in mm [28].

The existence of spondylophytes (bone spurs on the ventral circumference of the vertebrae) or dorsal spondylophytes (bone spurs on the dorsal circumference of the vertebrae) were recorded with yes/no responses.

Changes in the facies intervertebralis were classified according to Modic [29]. Three types of changes were considered. Modic type I shows oedematization as acute or ‘activated’ osteochondrosis. Type II presents as lipomatose degeneration of the facies intervertebralis, while type III presents as sclerosis of the facies intervertebralis.

Disc herniation is classified as ‘bulging’ (broad prominence affecting more than 25% of the disc), ‘protrusion’ (broader prominence but still with good contact to the disc), ‘extrusion’ (prominence with only little contact to the actual disc), and ‘sequester’ (disc material completely separated from the disc and isolated in the spinal canal).

The signal characteristics and structure were classified as ‘triple-layered’, ‘inhomogeneity’, and ‘signal attenuation’. Beforehand, we viewed multiple lumbar MRIs of younger patients in order to generate a baseline for normal disc signalling. To do so, the examination of a 20-year-old male patient with no degenerative changes was selected as a standard for comparison for the purposes of the study analysis.

Radicular compression can occur when disc material comes into contact with nerve roots. They are classified as ‘contact’, ‘displacement’, or ‘compression’, depending on their extent [30].

The extent of the spinal stenosis was classified as ‘relative’ or ‘absolute’. Relative spinal stenosis is the narrowing of the spinal canal with preserved cerebrospinal fluid (CSF) signal. Absolute spinal stenosis is present when no CSF signal can be detected. 

We deliberately decided against diagnosing arthritis of the facet joints in this project because consistent diagnostic criteria have yet to be developed. Figure 1 shows a typical sagittal T2-weighed MRI image of the lumbar spine. 

Previous analyses have revealed divergent findings with regard to inter-rater and intra-rater reliability of medical evaluation of lumbar MRIs [31,32]. In order to check the results of our investigation, following the compilation of the patient list and the full data entry for the entire study population in the analysis table, a further consultant radiologist reviewed another five patients in each age and sex category without being aware of the results of the primary reader. This meant that a total of 40 MRIs were assessed twice. In terms of content and form, this second assessment was done analogously to the primary assessment of all patients.

### 2.1. Statistical Analysis

We conducted descriptive analyses based on categorical variables with case figures and relative frequencies and the cohorts were compared using Fisher’s exact test where appropriate. Continuous values were analyzed using averages, presented in tabular form with the relevant standard deviations (SD) or 95% confidence intervals (95% CI) and compared using t-tests where appropriate. A Cochran Armitage test for trend in proportions was used to investigate the trend across the levels of an ordered variable. Binary logistic regression analyses were carried out with correction using the Firth method; dependent variables were, for example, ‘degeneration of the disc’s internal structures’ vs. ‘no degradation of the internal structures’. When selecting the independent variables, all risk factors for degeneration were taken into account. We also selected a binary logistic regression with ‘any degeneration of a disc’ as a parameter for studying the variables influencing the development of disc degeneration; mixed models were used here, where the subject was taken into account as a random factor so that test subjects were considered as a statistical unit. Potential influencing variables were modelled as fixed effects.

The percentage of congruent findings was calculated for inter-observer variability. A *p*-value of ≤0.05 was deemed statistically significant. The statistical analysis program R version 4.1.2 (R Foundation for Statistical Computing, Vienna, Austria) was used for the statistical analyses [33].

### 2.2. Technical Specifications of the MRIs

The examinations were carried out on a 1.5T Philips MRI scanner (Koninklijke Philips N.V., Amsterdam, Netherlands). T2-weighted turbo spin echo (TSE) sequences were conducted for each patient of the sagittal (FOV 160 × 320 mm, repetition time [TR] 3434 ms/echo, echo time {TE} 100 s) and transverse (FOV 150 × 200 mm, TR 3508 ms/echo, TE 120 ms) planes, as well as T1-weighted turbo spin echo sequences of the sagittal (FOV 160 × 320 mm, TR 490 ms/echo, TE 10 ms) plane. The slice thickness was 3 mm for each. The craniocaudal expansion comprised the area from at least L1 to L5. The transverse slices were carried out in areas of particular interest from a morphological imaging perspective following a radiological review.

## 3. Results

**Figure 2:**
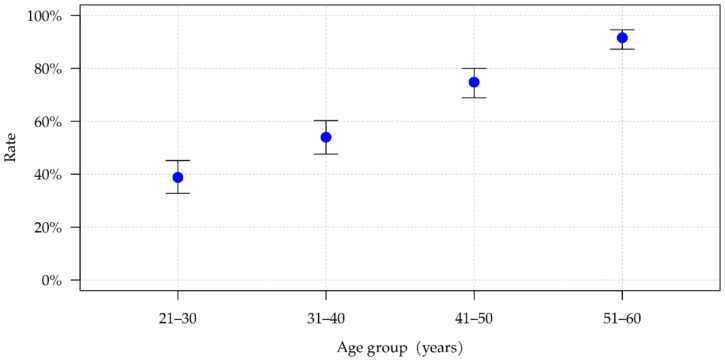
Percentage of degenerated disc segments per age group with relevant 95% confidence intervals.

A total of 1000 disc segments were assessed in 100 women and 100 men. This equaled 250 segments per age category. There were no statistically significant differences in the distribution of degenerative symptoms among men and women. For this reason, findings are not categorized by gender. Figure 2 shows the number of segments in which at least one of the seven degeneration characteristics is present. There is a practically linear association between age and number of segments showing signs of degeneration across the four investigated age groups (*p* < 0.001). Among 21- to 30-year-olds, 38.8% showed at least one symptom of degeneration, and this figure rose to 91.6% among 51- to 60-year-olds (Table 2). The mean number of positive signs of degeneration per segment with at least one positive sign of degeneration also increased with age. Once a segment has undergone degenerative changes, an average of 2.20 individual signs of degeneration can. Be found in the youngest age group, while 2.65 degenerative characteristics are seen within the oldest age group. This difference is statistically significant (*p* = 0.003).

**Figure 3:**
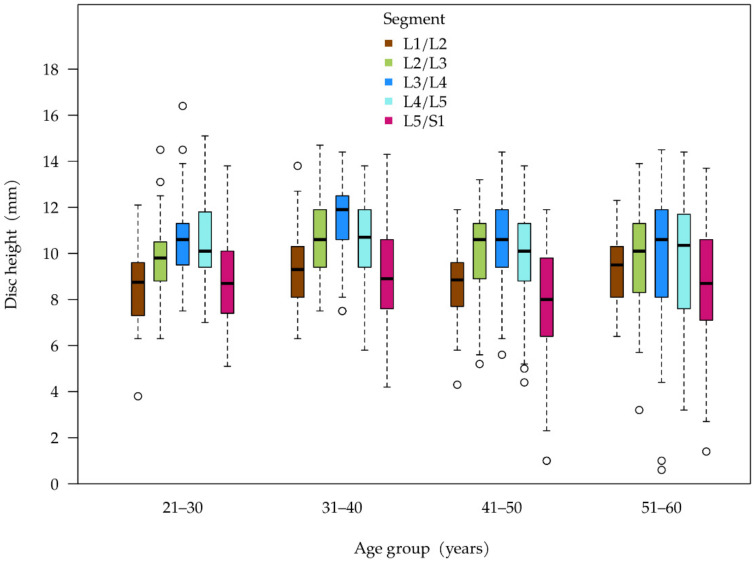
Disc height in mm per segment and age cohort.

Table 3 summarizes the disc height, structural changes, and herniation. There were no statistically significant variations in absolute disc height across all age groups (see Figure 3). In the assessment of disc height of individual segments, 60–64.5% of the relevant segments had a relative height of at least 80% across all age groups. There was a decrease in the segments with a relative height of 66–80% across all age groups (30% in cohort 1 to 21.5% in cohort 4)—with the most marked decrease between cohorts 1 and 2. Meanwhile, the proportion of discs with severely reduced height increased significantly. In cohort 1, with the youngest patients, two segments (1%) had a relative height of less than 50%, while in cohort 4, a total of 15 segments (7.5%) showed this kind of reduction in height (no table).

A similar distribution of disc height depending on the segment was seen across all age groups. The disc in segment L5/S1 has the lowest height and the disc in segment L3/L4 has the greatest height when seen as a median (Figure 3). What is noticeable is that the variability in disc height measured at the interquartile range (width of the boxes) increases with age, which corresponds to the increase in discs with a relative height of less than 50% (see above).

There was a statistically significant increase in segments with structural changes in the discs as age increases (*p* < 0.001) (Table 3). It is noteworthy that more than one-third (38.4%) of all discs in cohort 1 already show signs of structural changes. Furthermore, there is a statistically significant increase in the number of segments in which there is evidence of disc herniation (*p* < 0.001).

The assessment of the bone-related signs of degeneration also showed a significant increase across all age groups (Table 4). The formation of spondylosis is the most commonly observed osseous change. In the 51–60 age group, more than half (60.4%) of segments are affected. The formation of dorsal spondylophytes is, by contrast, rare even in the older age cohorts (6.4%). Osteochondrosis is just as rare in cohort 1 as spondylosis (each 2.4%). Osteochondrosis also becomes much more common in the older age groups. However, there is a significant difference in the oldest group of test subjects in terms of the frequency of spondylosis (60.4%) and osteochondrosis (23.6%).

In the second stage of the analysis, we evaluated the topic of segment degeneration. Table 5 shows the percentage of degenerated segments per intervertebral disc space for each age group, as well as the average number of positive degeneration characteristics.

The proportion of degenerated segments also increased with age in this representation method. In the cohort of the youngest test subjects, it was primarily the two caudal segments that were affected by degeneration. With increasing age, the cranial lumbar segments are more likely to be affected. In the cohort of the oldest subjects, the majority of segments were degenerated at all levels of the spine.

If the individual criteria for segment degeneration are also considered, the topical distribution with more common caudal degeneration is found in all age groups. The two caudal segments (L4/L5 and L5/S1) both have the most frequent positive individual criteria (Table 6). The most common degeneration criteria are changes to the disc’s internal structures. As with the other symptoms of degeneration, the lower two segments were the most commonly affected. For these two segments, however, there was no statistically significant correlation with age. Of the individual degenerative characteristics, nerve affection in particular and, partly, disc herniation in the individual segments, showed no statistically significant correlation between age and the number of degenerative symptoms.

In a further analysis, we evaluated the correlation between disc degeneration and osseous changes. All 1000 disc segments in the analysis were used as the database. For the presence of spondylosis, we calculated an OR of 1.46 (95% CI: 1.31; 1.62) per millimeter decrease in disc height. For the structural changes, we calculated an OR of 1.26 (95% CI: 1.16; 1.37) as compared to the triple-layering of the disc. For disc bulging, the OR came to 1.43 per mm decrease in disc height (Table 7).

As described under Method, we requested spot checks by requesting a second opinion from a different radiologist in order to check the validity of the MRI evaluations. This second radiologist evaluated a total of 40 of the 250 MRIs (10 MRIs for each age group) in accordance with the same criteria as had been applied for the first opinion.

In this second opinion, there were major similarities between both observers. For example, both agreed on ‘spinal stenosis’ in 99% of cases. The sub-points ‘nerve affection’ (95% concordance), ‘osteochondrosis’ (94.5% concordance), and ‘dorsal spondylosis’ (96% concordance) also showed practically no divergence.

There was a larger discrepancy in terms of the issue of internal structures. Here, there was a concordance of 77.5%. The initial assessor described a disc as being inhomogeneous (14.0%) or signal-attenuated (43.5%) much more frequently, while the second assessor more frequently stated the triple-layer structure (42.5% vs. 57.5%).

There was a similar tendency for the sub-point ‘spondylosis’, where there was consensus of 85.5%. In this point, the first assessor was more likely to describe spondylosis than the second assessor (24.0% vs. 15.5%).

## 4. Discussion

This study evaluated the degeneration patterns of a total of 1000 segments of the lumbar spine. The data obtained will help to support various pathophysiological models for lumbar segment degeneration. The hypothesis of the lumbosacral transitional discs as a zone of particularly early-onset and fast-progressing segment degeneration was once again confirmed. Segments L4/L5 and particularly L5/S1 are much more commonly affected by degenerative changes and, including in cases of multisegmental degeneration, also frequently show more progressive degeneration (more positive individual aspects of segment degeneration) than other segments.

Even in the early stage of study participant selection, we were able to identify initial indications of an age distribution of clinically relevant degeneration of the lumbar spine. Enrolling enough patients for the youngest cohort was the most challenging. In order to find the required number of patients for this age group, imaging data had to be evaluated from a 26-month period. By contrast, the 51–60 age group took nine months of data to complete, despite the fact that significantly more patients in this age group could not be enrolled due to exclusion criteria. This clearly shows the frequency distribution of the examinations in the various age groups and thus also the frequency of clinical symptoms.

In our study population, there was no statistically significant change across the age cohorts in terms of absolute disc height. Having said that, the analysis of relative disc height revealed a clear tendency towards an increased frequency of reduced-height segments in the older age groups. Furthermore, both cohorts of older subjects showed greater variation in individual disc heights.

Earlier studies have obtained partially contradictory data with regard to the correlation between disc height and age. For example, Videman et al. determined a decrease in disc height with advancing age in a longitudinal study [16]. Other studies also indicate a decrease in disc height with advancing age [34]. This corresponds to the decrease in relative disc heights determined in our study populations. In 2006, however, Pfirrmann et al. showed that decreasing disc height in advanced age only occurred when there were other symptoms of disc degeneration [35]. Our study population also showed a correlation between disc height and other characteristics of disc degeneration.

Earlier studies described correlations between disc height, patient height, and lifestyle [36,37]. Because it was not possible to collect data on these potential disruptive factors in our study design, we cannot rule out the possibility that our study population may be subject to a distortion in this regard. These modifying factors must therefore be taken into account in future studies.

Our data show that initial symptoms of disc degeneration begin to occur at a young age. As demonstrated in other studies, the structural changes to the disc, expressed as a change in signalling in the MRI, are the initial focus [35]. Decreased disc height and osseous degeneration processes only occur more frequently in the study cohorts of older patients.

It is noteworthy that in the segment L5/S1 the prevalence of disc structure impairment is stable between the analyzed age cohorts with already a 90% rate of disc structure impairment in the youngest age cohort. There was however a significant increase in the prevalence of spondylophytes and osteochondrosis. The possible link between early disc structure impairment and development of spondylophytes and osteochondrosis in these segments should be further investigated.

We were not able to determine any correlation between gender and lumbar segment degeneration. Earlier studies showed different results concerning gender and disc degeneration. There was no connection between both factors in a prospective study in 144 patients with low back pain [38]. Other studies suggested that women have more severe disc degeneration, albeit in higher age cohorts that we did not investigate [39].

It must be noted that our study only analyzed data from those patients with an indication of lumbar MRI. Based on our own clinical experience, by far the most common indication is lumbar pain due to degenerative changes. The study design did not have the scope to make statements about the extent to which there may be a gender difference between in terms of the indication.

We were not able to confirm the increasing frequency of spinal stenosis in advanced age [40] as described in the literature. It may be the case that the cut-off of 60 years that we selected was too low in order to be able to observe this effect of degeneration. On the other hand, we selected this age limit in order to be able to show degeneration patterns that could be expected among people of working age.

### Strengths/Weaknesses

With over 200 patients analyzed and seven distinctive individual aspects of lumbar segment degeneration studied, our results provide more conclusive information on the prevalence of degenerative processes of the lumbar spine. It is particularly noteworthy for the fact that it gathered information on both the disc-related and osseous degeneration processes, enabling us to make hypotheses about the correlations between these processes, which can be investigated in additional studies.

One limitation of our study is its retrospective nature. This meant that it was not possible to give image findings a clinical dimension by collecting data on symptoms. It also was not possible to gather patient medical histories. Because often no correlations, or only loose correlations, were found between lumbar pain and degenerative changes determined via morphological imaging, it would be desirable for a future study to adopt a design that would enable the patients’ potential symptoms to be recorded [11,41,42].

We consciously decided not to include the aspect of facet joint arthritis because this is, in our opinion, particularly difficult to assess. It was practically impossible to classify its extent, or to do so in such a way that comparable results could be determined by different assessors. Because the degeneration of the facet joints is both clinical and pathophysiologically an important aspect of lumbar segment degeneration [43], this should certainly be the subject of a subsequent study.

We consciously decided against the quantification of spondylosis or dorsal spondylosis. In our clinical experience, it is not currently possible to ensure the objective, reproducible and individually comparable measurement of spondylophytes.

## 5. Conclusions

Initial signs of lumbar segment degeneration frequently begin to occur in the 21–30 age group and primarily affect the structure of the disc. In study cohorts of older subjects, symptoms of concomitant osseous processes (osteochondrosis, spondylosis) are more visible. The segments that show disc degeneration are more likely to be affected by these sorts of processes. The disc segments most commonly affected by degeneration are L4/L5 and L5/S1.

With the aid of our study, we were able to establish an evaluation protocol for the cross-sectional study investigating occupational disc degeneration, which is now underway. This cross-sectional study will record additional data, particularly with regard to symptoms, lifestyle, occupational strain, and other illnesses. This data will facilitate a more detailed analysis, taking into account potential disruptive factors.

## Figures and Tables

**Table 1:** Composition of the study cohorts.

Age	Male	Female
21–30 years	25	25
31–40 years	25	25
41–50 years	25	25
51–60 years	25	25

**Table 2:** Frequency and extent of segment degeneration by age group (number of degenerated segments per age group and average positive individual markers of degeneration per segment).

Age Group	Degenerated Segments N (%)	Degeneration Characteristics Per Segment Average
21–30	72 (38.8)	2.20
31–40	135 (54.0)	2.04
41–50	187 (74.8)	2.55
51–60	229 (91.6)	2.65

**Table 3:** Extent and frequency of individual criteria for the assessment of disc degeneration (average corrected disc height, percentage of discs with structural changes [signal inhomogeneities or signal attenuation] and percentage of discs with prominence [bulging, protrusion, and extrusion]).

Age Group	Ø Corrected Disc Heights mm (SD)	Discs with Structural Changes Number (%)	Prominence/Bulging Number (%)
21–30	10.79 (2.02)	96 (38.4)	60 (24.0)
31–40	11.40 (2.14)	129 (51.6)	65 (26.0)
41–50	10.45 (2.42)	183 (73.2)	101 (40.4)
51–60	10.63 (2.78)	221 (88.4)	113 (45.2)

**Table 4:** List of osseous criteria for segment degeneration (number and proportion of segments with osteochondrosis, spondylosis and dorsal spondylosis for each age group).

Age Group	Osteochondrosis N (%)	Spondylosis N (%)	Dorsal Spondylosis N (%)
21–30	6 (2.4%)	6 (2.4%)	1 (0.4%)
31–40	24 (9.6%)	22 (8.8%)	2 (0.8%)
41–50	49 (19.6%)	81 (32.4%)	10 (4.0%)
51–60	59 (23.6%)	151 (60.4%)	16 (6.4%)

**Table 5:** Proportion of segments affected by degenerative processes, taking into account the individual segments and age of the subject with the average number of positive individual criteria for segment degeneration where at least one characteristic is positive.

	Age Group				
Discs	21–30 % (Mean)	31–40 % (Mean)	41–50 % (Mean)	51–60 % (Mean)	*p*_Trend, age_
L1/L2	14% (1.3)	26% (1.2)	54% (1.7)	78% (1.8)	<0.001
L2/L3	4% (1.0)	40% (1.3)	56% (1.8)	92% (2.3)	<0.001
L3/L4	20% (1.4)	38% (1.8)	72% (2.1)	96% (2.9)	<0.001
L4/L5	66% (2.5)	84% (2.3)	92% (3.1)	100% (3.1)	<0.001
L5/S1	90% (2.4)	82% (2.5)	96% (3.1)	96% (3.0)	0.083
*p*_Trend, segment_	<0.001	<0.001	<0.001	0.001	

**Table 6:** Number of individual degenerative characteristics by age group and segment (maximum number n = 50).

Degenerative Characteristic	Age Group					
	Disc	21–30 n	31–40 n	41–50 n	51–60 n	*p*_Trend, age_
Disc structure	L1/L2	7	12	26	34	<0.001
	L2/L3	2	18	28	43	<0.001
	L3/L4	10	19	35	48	<0.001
	L4/L5	32	39	46	50	<0.001
	L5/S1	45	41	48	46	0.299
	*p*_Trend, segment_	<0.001	<0.001	<0.001	<0.001	
Disc herniation	L1/L2	1	2	8	10	<0.001
	L2/L3	0	4	10	16	<0.001
	L3/L4	3	8	18	21	<0.001
	L4/L5	24	24	33	35	0.007
	L5/S1	32	27	32	31	0.898
	*p*_Trend, segment_	<0.001	<0.001	<0.001	<0.001	
Nerve affection,	L1/L2	0	0	1	0	0.729
	L2/L3	0	0	1	3	0.025
	L3/L4	0	0	6	5	0.003
	L4/L5	13	9	16	15	0.355
	L5/S1	25	19	19	17	0.123
	*p*_Trend, segment_	<0.001	<0.001	<0.001	<0.001	
Spinal stenosis	L1/L2	0	0	0	0	1.000
	L2/L3	0	0	0	1	0.274
	L3/L4	0	1	3	2	0.153
	L4/L5	4	3	2	4	0.901
	L5/S1	2	1	4	0	0.622
	*p*_Trend, segment_	0.020	0.124	0.017	0.435	
Osteochondrosis	L1/L2	0	1	4	5	0.009
	L2/L3	0	0	3	6	<0.001
	L3/L4	1	3	4	14	<0.001
	L4/L5	3	8	18	15	<0.001
	L5/S1	2	12	20	19	<0.001
	*p*_Trend, segment_	0.044	<0.001	<0.001	<0.001	
Spondylophytes	L1/L2	1	1	11	19	<0.001
	L2/L3	0	4	11	28	<0.001
	L3/L4	0	3	12	41	<0.001
	L4/L5	4	11	24	36	<0.001
	L5/S1	1	3	23	27	<0.001
	*p*_Trend, segment_	0.262	0.085	<0.001	0.029	
Dorsal spondylophytes	L1/L2	0	0	1	0	0.729
	L2/L3	0	0	1	4	0.007
	L3/L4	0	0	0	4	0.004
	L4/L5	1	2	3	4	0.156
	L5/S1	0	0	5	4	0.009
	*p*_Trend, segment_	0.577	0.375	0.023	0.150	

**Table 7:** Influence of the height of the disc segments (per mm decrease in disc segment) on the seven aspects of degeneration considered in the study.

Variable	Degenerated (N)	OR	95% CI	*p*
Disc structure	628	1.26	(1.16; 1.37)	<0.001
Disc herniation	339	1.43	(1.32; 1.54)	<0.001
Nerve affection	149	1.29	(1.19; 1.39)	<0.001
Spinal stenosis	27	1.24	(1.03; 1.50)	0.026
Osteochondrosis	138	2.04	(1.74; 2.39)	<0.001
Spondylophytes	260	1.46	(1.31; 1.62)	<0.001
Dorsal spondylophytes	29	2.52	(1.73; 3.76)	<0.001

## Data Availability

The data are available from the corresponding author upon request.
